# The Effects of Biologics on Hematologic Malignancy Development in Patients with Ankylosing Spondylitis, Psoriasis, or Psoriatic Arthritis: A National Cohort Study

**DOI:** 10.3390/biomedicines11092510

**Published:** 2023-09-11

**Authors:** Chia-Jung Tsai, Yu-Chih Lin, Chung-Yu Chen, Chih-Hsing Hung, Yi-Ching Lin

**Affiliations:** 1 Master Program in Clinical Pharmacy, School of Pharmacy, Kaohsiung Medical University, Kaohsiung 807, Taiwan; 2 Department of Pharmacy, Taipei Veterans General Hospital, Taipei City 112, Taiwan; 3 Department of Medical Humanities and Education, School of Medicine, Kaohsiung Medical University, Kaohsiung 807, Taiwan; 4 Division of Allergology, Immunology and Rheumatology, Department of Internal Medicine, Kaohsiung Medical University, Kaohsiung 807, Taiwan; 5 Department of Pharmacy, Kaohsiung Medical University Hospital, Kaohsiung 807, Taiwan; 6 Department of Medical Research, Kaohsiung Medical University Hospital, Kaohsiung Medical University, Kaohsiung 807, Taiwan; 7 Department of Pediatrics, School of Medicine, College of Medicine, Kaohsiung Medical University, Kaohsiung 807, Taiwan; 8 Research Center for Environmental Medicine, Kaohsiung Medical University, Kaohsiung 807, Taiwan; 9 Department of Pediatrics, Kaohsiung Medical University Hospital, Kaohsiung Medical University, Kaohsiung 807, Taiwan; 10 Department of Pediatrics, Kaohsiung Municipal Siaogang Hospital, Kaohsiung 807, Taiwan; 11 Department of Laboratory Medicine, Kaohsiung Medical University Hospital, Kaohsiung Medical University, Kaohsiung 807, Taiwan; 12 Department of Laboratory Medicine, School of Medicine, College of Medicine, Kaohsiung Medical University, Kaohsiung 807, Taiwan; 13 Doctoral Degree Program of Toxicology, College of Pharmacy, Kaohsiung Medical University, Kaohsiung 807, Taiwan

**Keywords:** biologics, hematologic malignancies, ankylosing spondylitis, psoriasis, psoriatic arthritis, autoimmune diseases

## Abstract

Biologics are used for ankylosing spondylitis (AS), psoriasis, and psoriatic arthritis (PsA) treatment. The association between biologics and the development of hematologic malignancies is controversial, and data on patients with AS, psoriasis, and PsA are scarce. This retrospective cohort study used data from 2010 to 2020 from Taiwan’s National Health Insurance Research Database (NHIRD). Patients with AS, psoriasis, and PsA were divided into a biologics and non biologics group after 1:10 propensity score matching. The hematologic malignancy incidences and the time-/dose-dependent effects on biologics were analyzed by Poisson regression to evaluate the incidence rate ratio (IRR). Of the 4157 biologics users and 38,399 non biologics users included in the study, 10 and 72 persons developed hematologic malignancies, respectively. Biologics only significantly increased the risk of hematologic malignancies in non-Hodgkin’s lymphoma (IRR: 2.48, 95% confidence interval (CI): 1.28–4.80). Different treatment patterns, types of biologics prescribed, cumulative defined daily doses, comorbidities, and comedications did not significantly affect hematologic malignancy development. A significantly increased risk was observed when biologics had been prescribed for 1–2 years (IRR: 2.95, 95% CI: 1.14–7.67). Clinical professionals should be aware of a patients’ risk of hematologic malignancies during the second year of biologic treatment.

## 1. Introduction

Autoimmune diseases have been reported to be associated with hematologic malignancies. Hematologic malignancy risks differ among various autoimmune diseases, including ankylosing spondylitis (AS) and psoriasis [1,2,3,4,5]. AS and psoriatic arthritis (PsA) have similar pathophysiologies and are classified as seronegative spondyloarthropathies [6]. The global prevalence rates of AS and psoriasis are 0.1–0.5% and 2%, respectively [7,8]. Psoriasis is a skin disease that causes red plaques and dry skin, and 20–30% of patients have psoriasis combined with PsA [9,10,11,12]. It has been reported that compared to the general population, patients with AS have higher risks of myeloma, and patients with psoriasis have a positive association with Hodgkin’s lymphoma, non-Hodgkin’s lymphoma, and melanoma [5,13].

AS, psoriasis, or PsA considerably impacts the patient’s functional capacity and quality, and the treatment goal for patients is to improve their quality of life [14]. For treatment-naïve patients, non biologics therapies, such as conventional synthetic disease-modifying antirheumatic drugs (csDMARDs), nonsteroidal anti-inflammatory drugs (NSAIDs), and glucocorticoids, are considered the first-line therapy for most autoimmune diseases [7,15,16]. Biologics are an alternative therapy for patients who have failed first-line therapy. Earlier use of biologics after traditional DMARDs become ineffective has been recommended after the introduction of a treat-to-target strategy for rheumatic diseases, which has shown its value in the quality of life, radiographic damage, and work stability [17,18,19]. However, the increasing use of biologics in the treatment of rheumatic diseases has raised concerns about the risk of different comorbidities including malignancies in autoimmune disease patients [20,21,22,23].

Previous studies have shown inconsistent results regarding the association between the use of biologics and the development of hematologic malignancies in patients with autoimmune diseases. In Morgan’s study, the risk of lymphoproliferative malignancies was significantly lower in RA patients treated with etanercept, a tumor necrosis factor α inhibitors (TNFi) biologic, than in csDMARD-treated patients [21]. In contrast, Calip’s study found that patients with RA, AS, or PsA treated with TNFi had an increased risk of non-Hodgkin’s lymphoma [22]. Kimball’s study of patients with psoriasis, on the other hand, found no increased risk of lymphoma for the use of TNFi biologics, including etanercept, adalimumab, and infliximab [23].

Not only are the risks of hematologic malignancy with biologics use disputed but also the data on patients with AS and PsA are scarce. Because RA is more prevalent than other autoimmune diseases, most studies have focused on the effect of biologics (e.g., TNFi) on patients with RA [20,21,22,24,25]. On the other hand, Taiwan’s National Health Insurance Research Database (NHIRD) has been a frequently used source of patient data for investigating various malignancy risks and disease treatment due to the population size [26]. Therefore, this study will use the NHIRD to investigate the risk of hematologic malignancy development and observe the time-/dose-dependent effects of biologics usage in AS, psoriasis, and PsA patients to provide more reliable real-world data.

## 2. Materials and Methods

### 2.1. Study Design

This retrospective cohort study examined the biologics exposure and risk of hematological malignancy development in patients with AS, psoriasis, and PsA using 2010–2020 data from Taiwan’s National Health Insurance Research Database (NHIRD), which covers more than 99% of the population in Taiwan. The NHIRD records disease diagnoses according to the International Classification of Diseases, Ninth Revision, Clinical Modification (ICD-9-CM) and ICD-10-CM. The Institutional Review Board of Kaohsiung Medical University approved this study.

### 2.2. Study Population

**Figure 1:**
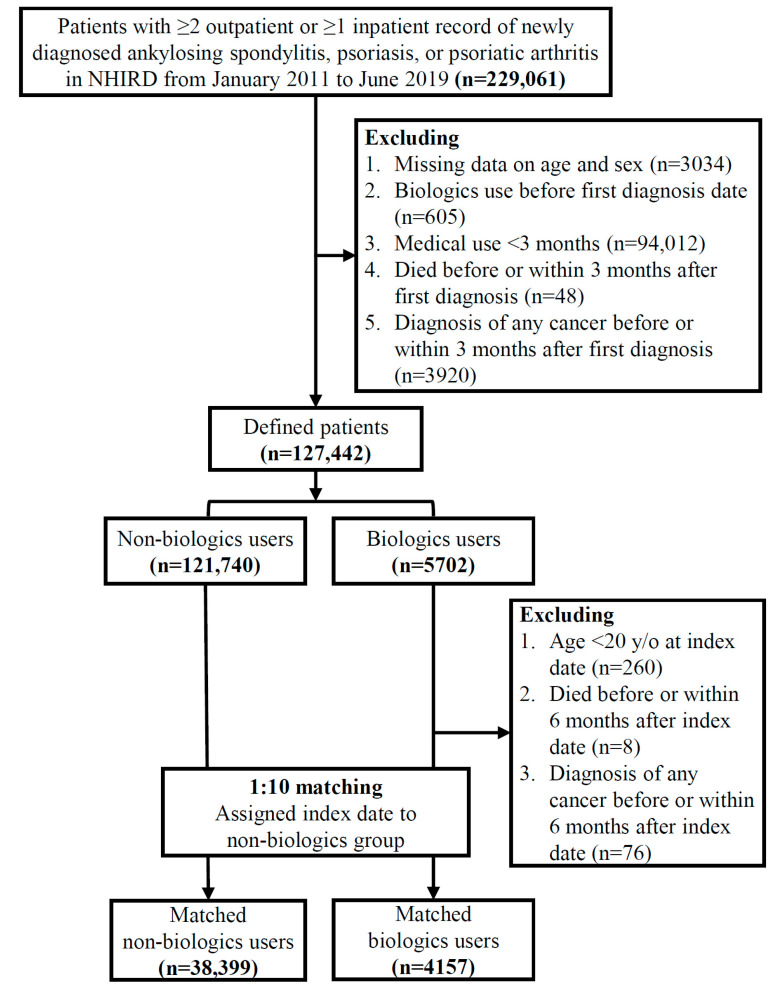
Flow chart of the patients selected for the cohort study.

Figure 1 shows the flow chart of the patients selected for the cohort study. Patients with more than two outpatient records or one inpatient record of newly diagnosed ankylosing spondylosis (ICD-9-CM/ICD-10-CM: 720.0/M45), psoriasis (ICD-9-CM/ICD-10-CM: 696.1/L40.0, L40.1, L40.2, L40.3, L40.4, L40.8, L40.9), and psoriatic arthritis (ICD-9-CM/ICD-10-CM: 696.0/L40.5) from January 2011 to June 2019 were eligible for inclusion in this study. Patients who had been treated for less than three months or patients who died or developed any cancer before or within three months after the first diagnosis date of AS, psoriasis, or PsA were excluded.

AS, psoriasis, and PsA patients treated with biologics approved in Taiwan before June 2019 were enrolled in the biologics group and the index date was defined as the date of the first prescription of biologics. Patients who used biologics before the first diagnosis date of AS, psoriasis, and PsA were excluded. The biologics included TNFi (etanercept, adalimumab, golimumab, infliximab, certolizumab, and opinercept), interleukin-6 (IL-6) inhibitors (tocilizumab), IL-12/23 inhibitors (ustekinumab and guselkumab), IL-17 inhibitors (secukinumab, ixekizumab, and brodalumab), rituximab, abatacept, and vedolizumab. We included all biologics, even those not explicitly approved for treating AS, psoriasis, and PsA, to validate the study population according to whether the correct biologics were used. AS, psoriasis, and PsA patients treated with non biologics, such as csDMARDs and NSAIDs, were enrolled in the non biologics group. Propensity score matching used 1:10 matching in the biologics group by sex, autoimmune disease indication, birth year, and year of first diagnosis of the disease, with the index date of the matching non biologics group being defined as the same dates as the matching biologic patients. Patients who died or developed cancer before or within six months after the index date were excluded in study population.

### 2.3. Outcomes

Patients with more than two outpatient codes or one inpatient diagnostic code for any hematologic malignancy in the follow-up period were defined as having outcome occurrences. The follow-up period began from the index date to the date that hematologic malignancy occurred, the patient died, was first diagnosed with cancer, or the study end date (December 2020), whichever came first. We set a six-month lag period to identify the effect of medication treatment.

Hematologic malignancies were categorized into lymphoid and myeloid malignancies. Lymphoid malignancies included lymphoid leukemia, multiple myeloma, immunoproliferative neoplasms, lymphoma (Hodgkin’s lymphoma and non-Hodgkin’s lymphoma), and other specified malignant tumors of the lymphatic tissue. Myeloid malignancies included myeloid leukemia, monocytic leukemia, and other specified leukemias (Table S1).

### 2.4. Covariates

Multivariate stratified analyses of baseline characteristics were conducted to observe the risk of hematologic malignancies in different strata. Patients’ age, sex, autoimmune disease duration at index, autoimmune disease indication, comorbidities, and comedication at baseline were analyzed. A medication used over three months was defined as a comedication. The comedications analyzed included the number of csDMARDs, analgesics, and corticosteroids. Analgesics have been reported to possibly have protective effects against hematologic malignancies [27,28].

### 2.5. Statistics

The analysis was performed using standardized differences for categorical and continuous variates. Standardized differences of more than 0.1 indicated that variates were clinically meaningful between the two study groups. The outcomes were analyzed by Poisson regression and adjusted by autoimmune disease indication, comorbidities, and comedications.

To identify the effect of biologics in different clinical treatment patterns, a comparison was conducted by subgroup among non biologics only, biologic monotherapy, and combination therapy. Patients were divided into non biologics only, TNFi biologics, and non-TNFi biologics groups to evaluate the type of biologics prescribed. Patients who used multiple types of biologics during the observation period were classified based on the biologics used for the “longest duration”. The cumulative defined daily dose (cDDD) is the total exposure to the defined daily dose, the assumed average maintenance dose per day for a drug, in a certain period. The cumulative time effect and dose–response effect of biologics were analyzed on the basis of the total duration of biologics prescription and the cDDD of biologics in the follow-up period.

Sensitivity analyses were performed to verify the reliability of our primary outcome. We compared our results for patients who had used biologics for at least three months with results for different periods: ever used, used less than one month, more than six months, and more than 12 months. We compared our event occurrence lag period of 6 months to lag periods of 3 months and 12 months. We verified the consistency of hematologic malignancy diagnoses between the NHIRD and data from the Taiwan Cancer Registry.

In statistical significance testing, significance levels of 0.05 (α = 0.05) for two-tailed tests were defined. The results are presented as the incidence rate ratio (IRR) with 95% confidence intervals and *p* values. All of the analyses were performed by the SAS software, version 9.4.

## 3. Results

### 3.1. Study Cohort and Patient Characteristics

We identified 229,061 patients with AS, psoriasis, or PsA from the NHIRD between January 2011 and June 2019. On the basis of the exclusion criteria, 101,619 patients were excluded before matching. After matching, 4157 and 38,399 patients were enrolled in the biologics group and the non biologics group, respectively (Figure 1). The mean age ± standard deviation (SD) of patients at the index date was 45.3 ± 15.1 years, and 65.6% of the patients were male.

We used PSM to select non biologics users as the reference group for biologics users. Most variables, including age, sex, and comorbidities, were similar between the biologics and non biologics groups. However, compared with the non biologics group, more patients in the biologics group were diagnosed with PsA, as well as using corticosteroids, and csDMARDs (Table 1). PSM calculated the sum of the probabilities of the matching variables. PsA might have relatively fewer subjects and a low weight percentage in the PSM, resulting in an imbalance after PSM. We adjusted these imbalanced variables in all the analyses to decrease the covariates’ effects between the biologics and non biologics groups.

### 3.2. The Risk of Hematological Malignancies between the Biologics Cohort and Non Biologics Cohort

Eighty-two overall hematologic malignancies were reported, including ten cases in the biologics group and seventy-two cases in the non biologics group. The median (interquartile range) follow-up time was 3.33 (3.63) years, and the incidence of hematologic malignancies was 643 per 106 person-years and 503 per 106 person-years in the biologics and non biologics groups, respectively. Patients treated with biologics did not have a significantly increased risk of overall hematologic malignancies and myeloid malignancies. However, the risks of lymphoid malignancies (IRR: 1.78, 95% CI: 1.08–2.93, *p* = 0.02), lymphoma (IRR: 2.78, 95% CI: 1.50–5.18, *p* < 0.01), and non-Hodgkin’s lymphoma (IRR: 2.48, 95% CI: 1.28–4.80, *p* = 0.01) were significantly higher in patients treated with biologics (Table 2).

### 3.3. The Risk of Hematological Malignancies between Different Treatment Patterns, Type of Biologics Prescribed, Duration of Biologics Prescription, and cDDD in Patients

Table 3 summarizes the subgroup analyses of different treatment patterns, types of biologics prescribed, duration of biologics prescription, and cDDD. Among patients treated with biologics, 78.4% received a combination therapy of biologics and non biologics, and 30% received TNFi. Compared to patients treated with non biologics, the risks of overall hematologic malignancies were not significantly different among different treatment patterns and different types of biologics prescribed.

Patients prescribed biologics had an elevated risk of hematologic malignancies during the second year. Nevertheless, there was no increased risk in patients prescribed biologics for less than one year or more than two years. There was no significantly increased incidence of newly developed hematological malignancies in the biologics group compared to the non biologics group at low (cDDD < 365), moderate (cDDD 365–1094), or high doses (cDDD ≥ 1095) (Table 3).

### 3.4. Possible Risk Factors for Hematological Malignancies in Biologics Users

The effects of biologics on the overall risk of hematologic malignancies were not significantly different in all variables examined using multivariate stratified analyses, which adjusted for autoimmune disease indication, comorbidities, and comedications. The IRRs were increased but without a significant difference in patients aged between 20 and 44 years at diagnosis, patients aged ≥65 years old either at diagnosis or at the index date, females, autoimmune disease diagnoses for <1 or 3–4 years at the index date, AS as an indication, hypertension, more than two csDMARDs prescribed, and without combined corticosteroid use (Table 4). 

### 3.5. Sensitivity Analyses

The primary outcomes shown in Table S2 are the data from more than three months of biologics use within a six-month lag period. The primary outcomes of hematologic malignancy development were consistent under different definitions of medication use (i.e., ever used, used more than one month, six months, or 12 months) and different lag periods of three and 12 months (Table S2). The adjusted IRR was still not statistically significant when the data of hematologic malignancy diagnoses were from the Taiwan Cancer Registry. (Table S3).

## 4. Discussion

This study investigated the risk of hematologic malignancy development and observed the time-/dose-dependent effects of biologics usage in AS, psoriasis, and PsA patients. Biologics did not increase hematologic malignancy development in patients with AS, psoriasis, or PsA in this retrospective cohort study except for non-Hodgkin’s lymphoma. The results were consistent before and after adjusting for the imbalance in baseline characteristics. The effects on hematologic malignancy development were not significantly different in treatment patterns, types of biologics prescribed, and cDDD. The risk of developing hematologic malignancy increased significantly only during the second year of biologics usage.

Some previous studies investigated the effects of biologics on hematological malignancy development in AS, psoriasis, or PsA [22,23,24,25,29]. In Calip’s study, patients with RA, AS, or psoriasis treated with TNFi had a higher risk of non-Hodgkin’s lymphoma, but a high proportion of patients diagnosed with RA (87.4%) resulted in limited data and a small population of patients with PsA and AS [22]. Our results are similar to the findings in Calip’s study; for our study population, we also found a higher risk of non-Hodgkin’s lymphoma with biologic treatment. Hellgren’s study assessed overall cancer risks and common subtypes in patients with spondyloarthritis, including AS and PsA, treated with TNFi [29]. The individuals who did not receive any treatment were defined as the controls in Hellgren’s study, and the data showed that treatment with TNFi was not associated with increased cancer risks. The reference group selection in Hellgren’s study might be unrealistic because most spondyloarthritis patients treated without biologics often received other therapies (e.g., csDMARDs, NSAIDs, etc.).

Because previous studies focused primarily on RA patients due to the relatively higher prevalence [24,25], we focused on patients with AS, psoriasis, and PsA in this retrospective cohort study. We found that biologics therapy was associated with a higher risk of non-Hodgkin’s lymphoma in patients with AS, psoriasis, or PsA. The increased risk for non-Hodgkin’s lymphoma development in biologics users may be due to the anti-immune effects of biologics, including the inhibition of NK cells and anti-lymphoma activity [30]. However, it is difficult to draw a definitive conclusion from these data due to the low incidences (n ≤ 5) of non-Hodgkin’s lymphoma in the biologic treatment group.

Higher proportions of patients with prior non biologics medication for treating their autoimmune disease were found in the biologics group. Under Taiwan’s National Health Insurance (NHI) program, AS, psoriasis, or PsA patients are reimbursed for biologics only after failing more than two kinds of non biologics therapy. This may account for the imbalance of medication between the biologics and non biologics groups. After adjusting for the variate imbalance, there was no significant difference among the subgroups of cDDD of biologics usage in patients with AS, psoriasis, or PsA. However, we found that for patients prescribed biologics, there was a higher risk of hematologic malignancies during the second year. A previous study also found a higher risk of non-Hodgkin’s lymphoma in patients with RA, AS, or PsA who used TNFi for 1.5–2.5 years [22]. There are some possible reasons for the U-shaped dose–response relationship between the duration of biologics use and the risk of hematologic malignancies: (1) the follow-up time for patients who used biologics was too short for malignancies to develop; (2) more frequent follow-up in the biologics group due to more severe disease activity might result in higher incidence and earlier discovery of hematological malignancies [22]; and (3) biologics might facilitate the growth of existing tumors [31]. Further investigation is necessary to explore these hypotheses and possible mechanisms.

This study had some limitations. First, the NHIRD database does not record clinical information not associated with insurance, such as laboratory results, personal or family history, and disease severity. To reduce the possible effect of disease severity, this study matched and adjusted for disease duration and comedication use for autoimmune diseases as surrogate variables for disease severity. Second, some factors related to hematologic malignancy development were not provided in the database, such as smoking, family history, and genetics. Third, because drug compliance is unknown, the cDDD might be overestimated.

Despite the limitations, our study had some strengths. Due to the low incidence rate of autoimmune diseases and hematologic malignancies, a large sample size is needed to identify the effects of biologics on hematologic malignancy development in autoimmune diseases. Because the NHIRD covers over 99% of the entire population in Taiwan, it provided a large sample size even after propensity score matching. All the patients in this study had received biologics or non biologic treatment for at least three months. This strict inclusion criterion might clarify whether the hematologic malignancy events were associated with the effect of the medications or with the underlying autoimmune diseases. In addition, compared to previous studies, this study investigated more detailed subtypes of hematologic malignancies and biologics dose–response effects, including the duration of the prescription and cDDD.

In summary, the use of biologics does not generally increase the risk of hematological cancers, except for non-Hodgkin’s lymphoma in patients with AS, psoriasis, and PsA. The risk of developing hematologic malignancy only significantly increased during the second year of biologics usage. This study, which focused on AS, psoriasis, and PsA patients, had a large sample size and included more recent biologics data. The criteria for selecting the study group were also more appropriate. These findings should alert healthcare professionals to the possible impact of biologics on the development of hematologic malignancies in patients with AS, psoriasis, and PsA and encourage further research into the mechanisms involved.

## Figures and Tables

**Table 1:** The baseline characteristics between the biologics cohort and non-biologics cohort.

Characteristics	Biologics (n = 4157)	Non-Biologics (n = 38,399)	StDiff
**Age at diagnosis, mean (SD)**	42.4 (15.4)	42.4 (15.3)	0.0005
**Age group at index, mean (SD)**	45.3 (15.2)	45.3 (15.1)	0.0008
45 > age ≥ 20	2098 (50.5%)	19,451 (50.7%)	0.0037
65 > age ≥ 45	1578 (38.0%)	14,589 (38.0%)	0.0007
Age ≥ 65	481 (11.6%)	4359 (11.4%)	0.0069
**Female, n (%)**	1444 (34.7%)	13,180 (34.3%)	0.0087
**AD duration at index, mean (SD)**	2.9 (2.3)	2.9 (2.3)	0.0001
**AD indication, n (%)**			
Ankylosing spondylitis	2332 (56.1%)	22,029 (57.4%)	0.0256
Psoriasis	1867 (44.9%)	16,032 (41.8%)	0.0638
Psoriatic arthritis	973 (23.4%)	6942 (18.1%)	0.1317 *
**Comorbidity, n (%)**			
Hypertension	985 (23.7%)	8145 (21.2%)	0.0595
Hepatitis B virus	144 (3.5%)	1015 (2.6%)	0.0477
Hepatitis C virus	62 (1.5%)	476 (1.2%)	0.0217
**Comedication, n (%)**			
Number of csDMARDs			
0	543 (13.1%)	19,625 (51.1%)	0.8925 *
1	2331 (56.1%)	14,598 (38.0%)	0.3679 *
≥2	1283 (30.9%)	4176 (10.9%)	0.5075 *
Analgesics	4088 (98.3%)	38,156 (99.4%)	0.0966
Corticosteroids	1105 (26.6%)	2234 (5.8%)	0.5873 *

*: Standardized difference > 0.1, considered clinically meaningful. n: number of the patients; StDiff: standardized difference; SD: standard deviation; AD: autoimmune disease; csDMARDs: conventional synthetic disease-modifying anti-rheumatic drugs.

**Table 2:** The risk of hematological malignancies between the biologics cohort and non-biologics cohort.

	Events		Crude IRR (95% CI)	*p* Value	Adjusted IRR ^†^ (95% CI)	*p* Value
	Biologics (n = 4157)	Non-Biologics (n = 38,399)				
**Overall hematologic malignancies**	10	72				
**Lymphoid malignancies**	8	43	2.80 (1.81, 4.35)	<0.01 *	1.78 (1.08, 2.93)	0.02 *
Lymphoid leukemia	0	≤5	NA	NA	NA	NA
Multiple myeloma and immunoproliferative neoplasms	≤5	16	1.99 (0.82, 4.80)	0.13	1.23 (0.46, 3.28)	0.67
Lymphoma	≤5	22	3.79 (2.19, 6.57)	<0.01 *	2.78 (1.50, 5.18)	<0.01 *
Hodgkin’s lymphoma	≤5	≤5	3.09 (0.62, 15.32)	0.17	NA	NA
Non-Hodgkin’s lymphoma	≤5	19	3.81 (2.13, 6.81)	<0.01 *	2.48 (1.28, 4.80)	0.01 *
Other specified malignant tumors of the lymphatic tissue	0	≤5	NA	NA	NA	NA
**Myeloid malignancies**	≤5	29	1.13 (0.45, 2.86)	0.79	1.06 (0.40, 2.86)	0.91
Myeloid leukemia	≤5	13	1.64 (0.48, 5.58)	0.43	1.48 (0.40, 5.43)	0.56
Monocytic leukemia	0	0	NA	NA	NA	NA
Other specified leukemias	0	16	NA	NA	NA	NA

*: *p* value < 0.05. ^†^: Adjusted for autoimmune disease indication, comorbidities, and comedications. IRR: incidence rate ratio; n: number of patients; CI: confidence interval; NA: not available.

**Table 3:** The risk of hematological malignancies among treatment patterns, types of biologics prescribed, duration of biologics prescription, and cDDD of biologics.

	E	n	PY	Crude IRR (95% CI)	*p* Value	Adjusted IRR ^†^ (95% CI)	*p* Value
**Treatment pattern**							
Non-biologics only	72	38,399	143,068	1.00 (Ref)		1.00 (Ref)	
Biologics monotherapy	≤5	898	2313	1.72 (0.42, 7.00)	0.45	1.56 (0.36, 6.80)	0.55
Biologics + non-biologics	≥6 ^‡^	3259	13,112	1.21 (0.58, 2.52)	0.61	0.75 (0.34, 1.65)	0.47
**Type of biologics prescribed**							
Non-biologics only	72	383,99	143,068	1.00 (Ref)		1.00 (Ref)	
TNFi	≤5	1250	3930	2.02 (0.74, 5.53)	0.17	1.42 (0.47, 4.32)	0.53
non-TNFi	≥6 ^&^	2907	11,495	1.04 (0.45, 2.39)	0.93	0.67 (0.28, 1.61)	0.38
**The total duration of biologics prescription**							
Non-biologics only	72	38,399	143,068	1.00 (Ref)		1.00 (Ref)	
<1 year	≤5	800	1282	1.55 (0.22, 11.15)	0.66	0.97 (0.13, 7.18)	0.98
1–2 years	≤5	895	2124	4.68 (1.89, 11.58)	<0.01 *	2.95 (1.14, 7.67)	0.03 *
2–3 years	≤5	836	2770	1.43 (0.35, 5.85)	0.61	1.04 (0.25, 4.40)	0.95
3–4 years	≤5	523	2237	0.89 (0.12, 6.39)	0.91	0.58 (0.08, 4.23)	0.59
≥4 years	≤5	1103	7013	0.28 (0.04, 2.04)	0.21	0.18 (0.03, 1.35)	0.10
**cDDD of biologics**							
Non-biologics only	72	38,399	143,068	1.00 (Ref)		1.00 (Ref)	
<365	≤5	793	1253	1.59 (0.22, 11.42)	0.65	1.00 (0.14, 7.42)	1.00
365–730	≤5	888	2147	3.70 (1.35, 10.13)	0.01 *	2.33 (0.81, 6.71)	0.12
731–1094	≤5	883	3010	2.64 (0.96, 7.23)	0.06	1.84 (0.64, 5.25)	0.26
≥1095	≤5	1593	9016	0.22 (0.03, 1.59)	0.13	0.15 (0.02, 1.07)	0.06

*: *p* value<0.05. ^†^: Adjusted for autoimmune disease indication, comorbidities, and comedications. ^‡^: The sum of the hematologic malignancy events in biologics monotherapy and biologics + non-biologics groups was 11. ^&^: The sum of the hematologic malignancy events in TNFi biologics and non-TNFi biologics groups was 11. cDDD: cumulative defined daily dose; IRR: incidence rate ratio; E: events of hematologic malignancies; n: number of the patients; PY: person years; CI: confidence interval; Ref: as the reference; TNFi: tumor necrosis factor-α inhibitors.

**Table 4:** Multivariate analyses of the risk of hematologic malignancies.

	Events of Hematologic Malignancies		Adjust IRR (95% CI) *	*p* Value
	Biologics	Non-Biologics		
**Age at diagnosis (years)**				
20 ≤ age < 45	5	32	1.06 (0.39, 2.89)	0.91
45 ≤ age < 65	≤5	28	0.46 (0.13, 1.64)	0.23
65 ≤ age	≤5	12	2.52 (0.54, 11.77)	0.24
**Age at index (years)**				
20 ≤ age < 45	4	34	0.81 (0.27, 2.43)	0.71
45 ≤ age < 65	≤5	28	0.50 (0.14, 1.81)	0.29
65 ≤ age	≤5	10	3.12 (0.7, 13.98)	0.14
**Sex**				
Male	6	54	0.70 (0.29, 1.73)	0.45
Female	4	18	1.16 (0.34, 3.90)	0.81
**AD duration (years)**				
duration ≤ 1	≤5	22	2.02 (0.71, 5.75)	0.19
1 < duration ≤ 2	≤5	27	0.35 (0.08, 1.61)	0.18
2 < duration ≤ 3	0	8	NA	NA
3 < duration ≤ 4	≤5	10	2.67 (0.66, 10.87)	0.17
4 < duration	0	5	NA	NA
**AD indication: AS**				
No	≤5	34	0.59 (0.17, 2.06)	0.41
Yes	≥6 ^†^	38	1.28 (0.52, 3.15)	0.59
**AD indication: PS**				
No	6	32	1.59 (0.62, 4.09)	0.34
Yes	4	40	0.57 (0.19, 1.70)	0.31
**AD indication: PsA**				
No	≥6 ^†^	58	1.06 (0.47, 2.37)	0.89
Yes	≤5	14	0.52 (0.11, 2.46)	0.41
**HTN**				
No	5	53	0.66 (0.25, 1.75)	0.41
Yes	5	19	1.23 (0.40, 3.79)	0.72
**HBV**				
No	≥6 ^†^	≥68 ^‡^	0.81 (0.38, 1.73)	0.59
Yes	≤5	≤5	0.99 (0.07, 14.02)	0.99
**HCV**				
No	10	72	0.84 (0.41, 1.73)	0.65
Yes	0	0	NA	NA
**Number of csDMARDs**				
<2	≤5	50	0.60 (0.18, 2.03)	0.41
≥2	≥6 ^†^	22	1.08 (0.43, 2.69)	0.87
**Analgesics**				
No	0	0	NA	NA
Yes	10	72	0.84 (0.41, 1.73)	0.63
**Corticosteroids**				
No	≥6 ^†^	62	1.44 (0.70, 2.99)	0.32
Yes	≤5	10	0.17 (0.02, 1.35)	0.09

*: Adjusted for autoimmune disease indication, comorbidities, and comedications. ^†^: The sum of the hematologic malignancy events in the biologics group was 11. ^‡^: The sum of the hematologic malignancy events in the non-biologics group was 72. IRR: incidence rate ratio; CI: confidence interval; AD: autoimmune disease; AS: ankylosing spondylitis; PS: psoriasis; PsA: psoriatic arthritis; HTN: hypertension; HBV: hepatitis B virus; HCV: hepatitis C virus; csDMARDs: conventional synthetic disease-modifying anti-rheumatic drugs. NA: not available.

## Data Availability

The data that support the findings of this study are available from Taiwan’s National Health Insurance Research Database (NHIRD). The data are not publicly available due to the “Personal Information Protection Act” in Taiwan. The datasets are available on request from the corresponding author.

## Supplementary Materials

The following supporting information can be downloaded at: https://www.mdpi.com/article/10.3390/biomedicines11092510/s1, Table S1: ICD-9-CM and ICD-10-CM codes of hematologic malignancies; Table S2: Sensitivity analysis of the risks of hematologic malignancies between biologics cohort and non-biologics cohort; Table S3: Sensitivity analysis of the data resources of hematologic malignancy diagnoses.
