# Supplementary material for: The Effects of Biologics on Hematologic Malignancy Development in Patients with Ankylosing Spondylitis, Psoriasis, or Psoriatic Arthritis: A National Cohort Study
Source: Biomedicines. 2023 Sep 11;11(9):2510. doi: 10.3390/biomedicines11092510 (PMC10526259; doi:10.3390/biomedicines11092510)
Supplement: Supplementary file 1 [file biomedicines-11-02510-s001.zip › biomedicines-2582876-supplementary.pdf]

Table S1. ICD-9-CM and ICD-10-CM codes of hematologic malignancies

	ICD-9-CM	ICD-10-CM
**Overall hematologic malignancies**	200-208	C81-C86, C88.2-C88.9, C90-C93, C94.0-C94.4, C94.8, C95, C96.0-C96.5, C96.9, C96.A, C96.Z, D45
**Lymphoid malignancies**	200, 201, 202, 203, 204	C81-C86, C88.2, C88.3, C88.4, C88.8, C88.9, C90, C91, C96.0-C96.5, C96.9, C96.A, C96.Z
Lymphoid leukemia	204	C91.0, C91.1, C91.2, C91.3, C91.5, C91.6, C91.9, C91.A, C91.Z
Multiple myeloma and immunoproliferative neoplasms	203	C88.2, C88.3, C88.8, C88.9, C90
Hodgkin's lymphoma	201	C81
Non-Hodgkin's lymphoma	202	C82, C83.1, C83.3, C83.8, C84.0, C84.1, C84.4, C84.9, C84.A, C84.Z, C85, C86.0, C86.1, C86.2, C86.3, C86.4, C88.4, C91.4, C96.0, C96.2, C96.4, C96.9, C96.A, C96.Z
Other specified malignant tumors of the lymphatic tissue	200	C83.0, C83.5, C83.7, C83.9, C84.6, C84.7, C86.5, C86.6, C96.5
**Myeloid malignancies**	205, 207, 208	C92, C93, C94.0, C94.2, C94.3, C94.8, C95, D45
Myeloid leukemia	205	C92
Monocytic leukemia	206	C93
Other specified leukemias	207, 208	C94.0, C94.2, C94.3, C94.8, C95, D45

ICD-9-CM: International Classification of Diseases, Ninth Revision, Clinical Modification; ICD-10-CM: International Classification of Diseases, Tenth Revision, Clinical Modification.

Table S2. Sensitivity analysis of the risks of hematologic malignancies between biologics cohort and non-biologics cohort

	Biologics			Non-biologics			Adjusted IRR*	
	E	n	PY	E	n	PY	(95% CI)	P value
**Primary outcome†**	10	4,157	15,426	72	38,399	143,068	0.84 (0.41, 1.73)	0.63
**Different lengths of Biologics use**								
Ever	9	4,435	16,182	73	41,751	153,109	0.72 (0.33, 1.56)	0.41
≥1 month	10	4,441	16,197	78	41,496	152,271	0.75 (0.36, 1.56)	0.44
≥6 months	10	3,785	14,679	87	34,473	134,337	0.77 (0.38, 1.55)	0.46
≥12 months	9	3,181	13,263	77	28,302	117,479	0.76 (0.37, 1.57)	0.46
**Lag period**								
3 months	16	4,149	15,321	82	38,517	142,182	1.25 (0.70, 2.26)	0.45
12 months	12	4,087	15,404	64	37,752	145,098	1.30 (0.66, 2.54)	0.45

*Adjusted for autoimmune disease indication, comorbidities, and comedications.

†Biologics use ≥3 months and observation period after 6 months.

E: events of hematologic malignancies; n: number of patients; PY: person-years; IRR: incidence rate ratio; CI: confidence interval.

Table S3. Sensitivity analysis of the data resources of hematologic malignancy diagnoses

	Biologics (n=4,157)		Non-biologics (n=38,399)		Adjusted IRR* (95% CI)	P value
	E	PY	E	PY		
NHIRD (primary outcome)	10	15,426	72	143,068	0.84 (0.41, 1.73)	0.63
Taiwan Cancer Registry	7	15,428	31	143,149	1.36 (0.56, 3.33)	0.50

*Adjusted for autoimmune disease indication, comorbidities, and comedications.

E: events of hematologic malignancies; n: number of patients; PY: person-years; IRR: incidence rate ratio; CI: confidence interval.
